# 3‐(3‐Hydroxyphenyl)‐Propionic Acid (PPA) Suppresses Osteoblastic Cell Senescence to Promote Bone Accretion in Mice

**DOI:** 10.1002/jbm4.10201

**Published:** 2019-08-23

**Authors:** Jin‐Ran Chen, Umesh D Wankhade, Alexander W Alund, Michael L Blackburn, Kartik Shankar, Oxana P Lazarenko

**Affiliations:** ^1^ Arkansas Children's Nutrition Center Little Rock AR USA; ^2^ Department of Pediatrics University of Arkansas for Medical Sciences Little Rock AR USA; ^3^ Graduate Program in Interdisciplinary Biomedical Sciences University of Arkansas for Medical Sciences Little Rock AR USA

**Keywords:** BONE FORMATION, BLUEBERRY, PHENOLIC ACID, PPARγ, G‐PROTEIN COUPLED RECEPTOR

## Abstract

Phenolic acids (PAs) are metabolites derived from polyphenolic compounds found in fruits and vegetables resulting from the actions of gut bacteria. Previously, we reported that the levels of seven individual PAs were found to be at least 10 times higher in the serum of rats fed a blueberry (BB)‐containing diet compared to those fed a control diet. We have characterized the effects of one such BB‐associated serum PA, 3‐(3‐hydroxyphenyl)‐propionic acid (PPA), on senescence signaling and promotion of mesenchymal stem cell differentiation toward osteoblasts, while suppressing adipogenesis in the stem cells. To better understand the mechanistic actions of PPA on bone formation in vivo, we administered four doses of PPA (0.1, 0.5, 1, and 5 mg/kg/day; daily i.p.) to 1‐month‐old female C57BL6/J mice for 30 days. We did not observe significant effects of PPA on cortical bone; however, there were significantly higher bone volume and trabecular thickness and increased osteoblastic cell number, but decreased osteoclastic cell number in PPA‐treated groups compared to controls. These morphological and cellular outcomes of bone were reflected in changes of bone formation markers in serum and bone marrow plasma. PPA treatment reduced senescence signaling as evaluated by senescence‐associated β‐galactosidase activity, PPARγ, p53, and p21 expression in bone. In conclusion, PPA is capable of altering the mesenchymal stem cell differentiation program and bone cell senescence. This raises the possibility that BB‐rich diets promote bone growth through increasing systemic PAs, a question that merits additional investigation. © 2019 The Authors. *JBMR Plus* published by Wiley Periodicals, Inc. on behalf of American Society for Bone and Mineral Research.

## Introduction

Bone development and remodeling are coordinately regulated by both local paracrine molecules and hormones travelling through the bloodstream.^1^, ^2^, ^3^ The human skeleton also requires an adequate supply of many different nutritional factors to optimize peak bone mass and minimize osteoporosis risk associated with normal aging. Of the individual nutritional factors, particular attention has been paid to micronutrients such as calcium, vitamin D, and phosphate, and macronutrients such as fat and protein. There has also been interest in several food groups, particularly dairy products, fruits and vegetables, and foods contributing to acid‐base balance, interacting with local bone transcription factors and circulating endogenous hormones to facilitate bone development.^4^, ^5^ These food‐based biologically active phytochemicals may not only promote bone development but also protect bone from degeneration. To date, the most investigated effects of phytochemicals on bone are phytoestrogens (ie, isoflavones found in soy)^6^ and resveratrol (found in red wine);^7^ whether they can exert significant effects on bone formation and produce greater bone quality in vivo still needs to be fully verified.

We have previously demonstrated a robust effect of blueberry (BB)‐supplemented diets on stimulating bone formation in male and female rodents.^8^ Such effects of BB on bone may be ascribed to higher levels of specific phenolic acids (PAs) appearing in the bloodstream of rodents after diets.^8^ Among those PAs, hippuric acid (HA) and 3‐(3‐hydroxyphenyl)‐propionic acid (PPA) were found to be potentially bioactive on stimulating osteoblast differentiation and proliferation in cell cultures.^9^ PAs are gut microflora–derived metabolites of polyphenols found in the circulation following consumption of BB and other fruits, vegetables, and coffee.^10^ PAs are structurally similar to nicotinic acid or niacin (the essential nutrient, vitamin B3); the latter is a nicotinic acid metabolite which binds and activates the G‐protein coupled receptor (GPR) 109 A.^11^ We have hypothesized that the actions of HA on bone cells is mediated in part through mechanisms involving binding to GPR109A in cell membranes.^11^ However, PPA actions on bone in vivo, and the potential mechanisms involved, remain to be elaborated further.

Bone formation is dependent on the differentiation and activity of osteoblasts; whereas resorption of preexisting mineralized bone matrix by osteoclasts is necessary for bone remodeling.^12^ In rapidly growing animals, active osteoblastic bone formation usually exceeds bone resorption, resulting in net bone accrual. Bone marrow stromal cells and periosteal osteoblast precursors are both potential sources of new osteoblasts.^3^ It is estimated that osteoblasts in mice have a lifetime of approximately 10 days,^13^ following which these cells are thought to be lost via apoptosis, become trapped in bone matrix giving rise to osteocytes, or become inactive lining cells. In addition to these processes, we have made the novel observations that increased osteoblastic cell senescence signaling may be fundamental to explain loss of osteoblast function and osteoprogenitor differentiation potential.^14^ Most important, we have discovered that biologically active dietary factors such as isoflavones and phenolic acids can inhibit senescence programs in osteoblasts or their precursor osteoprogenitors, which should promote bone development.^15^ Using approaches we have established previously, we set to test the hypothesis that PPA is able to suppress cellular senescence signaling in osteoblasts and bone both in vitro and in vivo.

## Materials and Methods

### Animals and PPA treatment

Twenty‐day‐old newly‐weaned female C57BL/6 J mice purchased from the Jackson Laboratory (Bar Harbor, Maine, USA) were randomly assigned to one of five groups (*n* = 10 per group). Mice are all weighed, then randomized due to their weight, and housed in mouse small shoe box cage, five animals per cage. One group of mice received AIN‐93G diet formulated with casein as the sole protein source throughout the experiment, and received a daily intraperitoneal injection of 100 µL saline (control). The other four groups of mice received a daily morning i.p. injection of 3‐(3‐hydroxyphenyl)‐propionic acid (PPA; Alfa Aesar, Haverhill, ME, USA; cas#621‐54‐5) 0.1, 0.5, 1, or 5 mg/kg/day made in saline for 30 days, designated as 0.1, 0.5, 1, and 5 PPA groups, respectively. These doses were based on serum PPA concentrations of rats fed a BB‐containing diet and in vitro PPA experiments in cell cultures.^8^ All four PPA treatment groups were pair‐fed to control, and their food and calorie intakes were matched to control group. Mice were housed in an Association for Assessment and Accreditation of Laboratory Animal Care–approved animal facility in the Arkansas Children's Nutrition Center Animal Studies Core at the Arkansas Children's Research Institute, with constant humidity and lights on from 6:00 a.m. to 6:00 p.m. at 22°C. All animal procedures were approved by the Institutional Animal Care and Use Committee at University of Arkansas for Medical Sciences (UAMS, Little Rock, AR, USA). After 30 days of injections (age postnatal day 55 [PND55]), mice were anesthetized by injection with 100 mg Nembutal/kg body weight (Avent Laboratories, MA, USA). Blood was collected via cardiac puncture, which was followed by decapitation; femur, tibia, and vertebrae were collected.

### µCT bone analyses

µCT measurements of the trabecular and cortical compartments from the right tibial bone were evaluated using a Scanco μCT scanner (μCT‐40; Scanco Medical AG, Bassersdorf, Switzerland) at 12 μm voxel resolution. The trabecular compartment included a 0.9 mm region extending distally 0.03 mm from the physis. All data were acquired at 70 kVp, 114 mA, and 200 ms integration time. The grayscale images were processed by using a low‐pass Gaussian filter (σ = 0.8, support = 1) to remove noise, and a fixed threshold of 245 was used to extract the mineralized bone from the soft tissue and marrow phase. Cancellous bone was separated from the cortical regions by semiautomatically drawn contours. A total of 120 slices starting from about 0.1 mm distal to the growth plate, 0.70 mm in length, were evaluated for trabecular bone structure, bone volume fraction (BV/TV, %), trabecular thickness (Tb.Th, mm), trabecular separation (Tb.Sp, mm), trabecular number (Tb.N, 1/mm), connectivity density (ConnD, 1/mm^3^), and structure model index (SMI) were calculated based on descriptions by Bouxsein and colleagues^16^ and Cao and colleagues,^17^ and by using software provided by Scanco, as described in detail previously.^17^, ^18^ For cortical bone of the tibia, the cortical compartment was a 0.6‐mm region extending distally starting 5 mm proximal to the tibia‐fibula junction. Total cross‐sectional area (CSA, mm^2^), medullary area (MA, mm^2^), and cortical thickness (Ct.Th, mm) were assessed.

### Bone histology and histomorphometry

Mouse lumbar vertebra (L_5_) samples were embedded, cut, and von Kossa and TRAPase‐stained by Histology Special Procedures, Department of Biology, School of Science, A Purdue University School, Indianapolis, IN, USA, and at histological core, Arkansas Children's Nutrition Center. For histomorphometric analysis, sections were read in a blinded fashion. Parameters of cancellous bones in the body of vertebra were measured with a digitizing morphometry system, which consists of an epifluorescent microscope (model BH‐2; Olympus), a color video camera, and a digitizing pad (Numonics; 2206) coupled to a computer (Sony) and a morphometry program OsteoMetrics (OsteoMetrics, Decatur, GA, USA). Total bone area, osteoblast number and surface, osteoclast number and surface, eroded surface, and other parameters were obtained by manual tracing as described previously.^8^

### Measurements of bone turnover markers in serum and bone marrow plasma

Blood serum and bone marrow plasma were prepared at the time of tissue harvest. Bone marrow was flushed out from femur using 300 µL PBS, vortexed, and spun (1700*g*) for collecting supernatant as bone marrow plasma. The serum and bone marrow plasma bone formation marker, bone‐specific alkaline phosphatase (ALP), was measured by Rat‐MID™ ALP ELISA, from Nordic Biosciences Diagnostic (Herlev, Denmark). The serum and bone marrow plasma total osteocalcin (Gla‐OC) and undercarboxylated osteocalcin (Glu‐OC) levels were measured by an ELISA‐based kit from TaKaRa Bio (Otsu, Japan). The serum bone resorption marker C‐terminal telopeptides of type I collagen (CTX‐1) was measured by Rat‐Laps™ ELISA from Nordic Biosciences Diagnostic (Herlev, Denmark).

### Cell cultures

Bone marrow–derived mouse stromal cell line ST2 cells were obtained from the Riken Cell Bank (Ibaraki, Japan), and mouse calvarial cells (isolated from 4‐day‐old male and female mouse pups after sequential collagenase digestion) were cultured in 75‐cm^2^ flasks in α‐MEM supplemented with 10% fetal bovine serum (FBS) (Hyclone, Logan, UT, USA), penicillin (100 Units/mL), streptomycin (100 µg/mL), and glutamine (4 mM). These cell cultures were previously described in our laboratory.^19^ ST2 cells were seeded in six‐well plates and treated with five different concentrations of PPA in the presence of either osteogenic medium or adipogenic medium. For the concentrations of PPA, 10 µg/dL is equivalent to the PPA level in animal blood after consumption of a 10% (of calories) blueberry diet, and referred to as 1 × for in vitro cell treatment; therefore, 0.1 ×  = 1 µg/dL, 0.5 ×  = 5 µg/dL, 5 ×  = 50 µg/dL, and 10 ×  = 100 µg/dL. Standard ALP activity staining was performed after 10‐day treatments, and Oil Red O staining was performed after 7‐day treatments in cell cultures according to methods published previously.^20^ In other experiments, cell RNA isolation was performed after cells were treated with PPA for 24 hours. To generate replicative senescent ST2 cells, we cultured 2 × 10^4^ cells/well in a six‐well plate; when the cells became confluent after 5 days, they were passaged. This procedure was repeated 29 times for a total of 30 passages with or without HA or PPA treatments. Methods of ST2 cells treated with 4 hours starvation (absence of serum) and 24 hours pretreatment with 25 µM hydrogen peroxide (H_2_O_2_) to induce cellular senescence were described previously.^21^

### ^35^S‐GTPγS binding assay

Ligand‐induced ^35^S‐GTPγS binding was studied using membrane fractions prepared from mouse fetal calvarial cells stably transfected with mouse GPR109A overexpression plasmid (OriGene, Rockville, MD, USA; catalog number RG206527) or GPR109B (OriGen Technologies; catalog number RG208523). Nicotinic acid used in this study was purchased from Sigma Chemical (St. Louis, MO, USA). Acifran was synthesized by chemists at Arena Pharmaceuticals (San Diego, CA, USA). ^35^S‐GTPγS (1160 Ci/mmol) was from Amersham Biosciences (Piscataway, NJ, USA). Binding of ^35^S‐GTPγS was determined in the presence of increased concentrations of nicotinic acid, acifran, HA, or PPA. The membranous, cytosolic, and nuclear fractions of proteins from cells were prepared according to the procedure provided by the manufacturer (Pierce Biotechnology, Rockford, IL, USA). Detailed procedure of binding assay was previously described by Ren and colleagues,^11^ and binding was allowed to proceed for 1 hour before centrifuging the plates at 4000 rpm (1700*g*) for 15 min at room temperature and subsequent counting in a TopCount scintillation counter.

### Senescence‐associated β‐galactosidase (SABG) activity assay

The SABG activity assay was performed using a β‐galactosidase enzyme assay kit (Promega, San Luis Obispo, CA, USA), measuring the absorbance at 420 nm according to the manufacturer's instructions. Isolation of proteins from cells and bone tissues for SABG activity measurements were described previously.^20^, ^22^

### RNA isolation, real‐time reverse‐transcription polymerase chain reaction

Cell and bone tissue RNA isolation from in vitro cultured cells and in vivo tissues were extracted using TRI Reagent (MRC Inc., Cincinnati, OH, USA) according to the manufacturer's recommendations followed by DNase digestion and column cleanup using QIAGEN mini columns (QIAGEN, Valencia, CA, USA).^22^ Reverse transcription was carried out using an iScript cDNA synthesis kit from Bio‐Rad (Hercules, CA, USA). All primers for real‐time PCR analysis used in this report were designed using Primer Express software 2.0.0 (Applied Biosystems, Foster City, CA, USA).

### RNA‐seq–based gene expression analysis

RNA‐seq was performed using RNA isolated from femurs from saline control and 0.5 mg/kg/day groups. Total RNA was isolated from femurs of individual mice (*n* = 10 per group). Poly‐A mRNA from each individual RNA sample (1 μg) was isolated using mRNA Direct reagents. Equal amounts of polyA‐mRNA from two to three mice were pooled, to generate three biologically distinct replicates per treatment group, representing all animals. Directional RNA‐seq libraries were prepared using NEBNext reagents as described previously^23^ and validated using electrophoresis and quantified using Qubit dsDNA reagents. Single‐read 75‐bp sequencing of libraries was performed using a NextSeq500 (Illumina, San Diego, CA, USA). Data analysis was performed via alignment of high‐quality reads using Bowtie2, and quantitation of read counts to genes using SeqMonk.^24^, ^25^ Differentially expressed genes were identified via DeSeq2 package in R ( ± 2‐fold change; false discovery rate [FDR] corrected *p* values < 0.05). The lists of differentially expressed genes were analyzed for GO biological process enrichment using the BiNGO plugin in Cytoscape. To specifically examine expression differences in bone‐related genes, we compiled a list of genes with known functions in bone and skeletal biology. Using the mouse genome informatics gene ontology reference (MGI; http://www.informatics.jax.org/), we identified 550 genes with terms “ossification,” “osteoblasts,” “osteoclasts,” “skeletal,” or “bone” in either one of three ontologies (Biological Process, Cellular component or Molecular functions). This list, referred to as bone‐related genes, were further analyzed for differential expression for a targeted analysis.

### Statistical analyses

Statistical power was computed based on a two‐factor ANOVA with 10 mice per group. In our preliminary analysis of the effects of 5% blueberry diet supplementation, or HA injection using a smaller sample size (8 rats/group, 5 mice/group) than in the proposed study, the European statistical forum (ES[f]) from comparing tibial cortical BMD between blueberry diet‐fed rats to those on normal chow was 0.89, whereas an ES(f) = 1.34 was observed when comparing tibial trabecular bone mineral density (BMD) between the two groups. Hence, with a sample size of 10/group, we should have sufficient power to detect meaningful differences. Numerical variables were expressed as means ± SE. Comparisons between groups were performed with the nonparametric Kruskal‐Wallis test followed by a Dunnett's test comparing each dose to the control group. The nonparametric Wilcoxon rank‐sum test was used to compare controls to individual treatment. Cell culture experiments were conducted at least three independent times, and representative images are displayed. Dose or time response was assessed using Cruick's nonparametric test for trend. The critical *p* value for statistical significance was *p* = 0.05.

## Results

### PPA stimulates osteoblast differentiation but inhibits adipogenesis in ST2 cells

**Figure 1:**
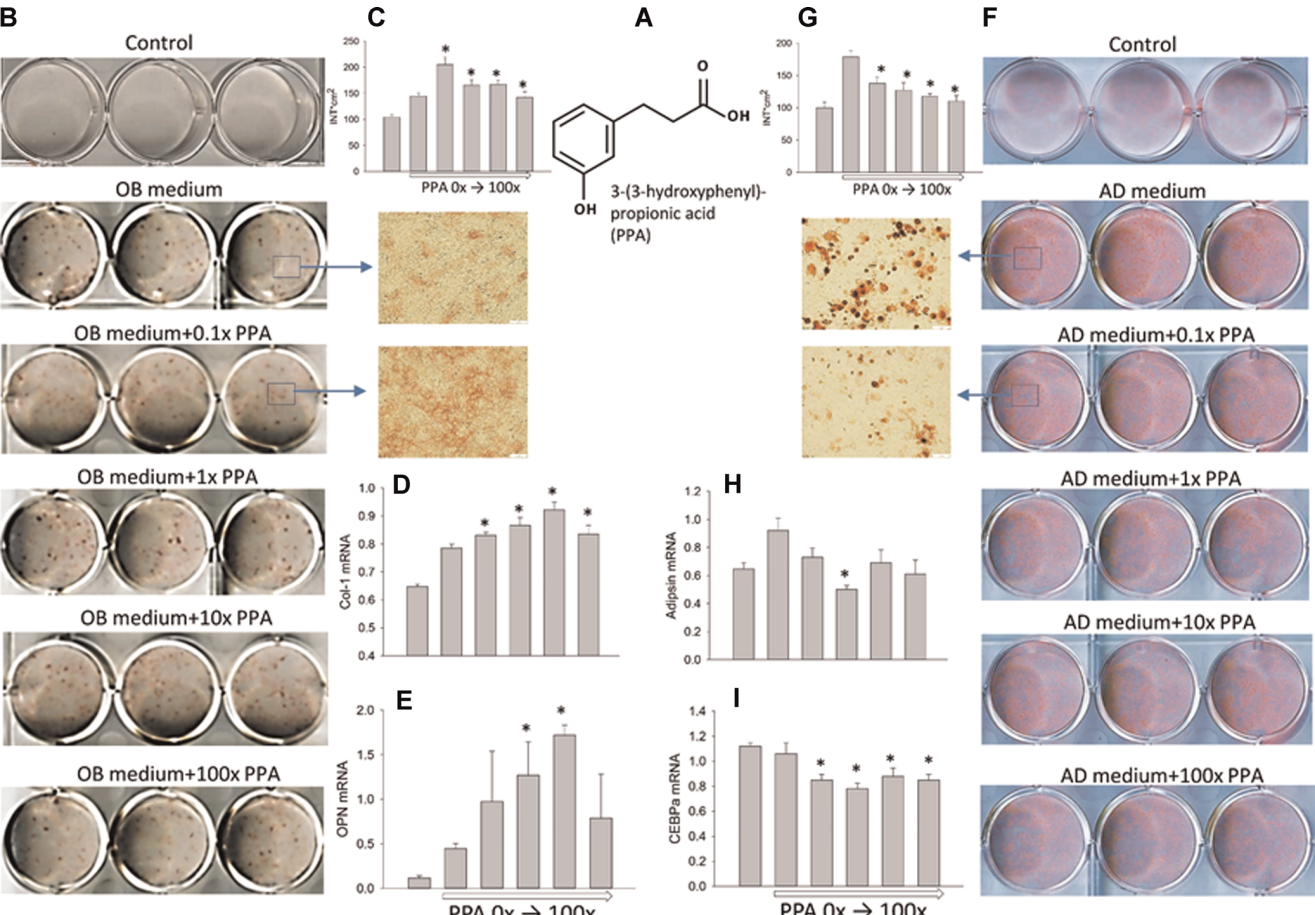
PPA stimulates in vitro osteoblastic cell differentiation but suppresses adipogenesis. (*A*) Structure of PPA we identified in serum of BB‐fed rats. (*B*) PPA stimulated the osteoblast differentiation evaluated by ALP staining after 10 days of cultures of ST2 cells; 1 × represents concentration found in serum of BB diet animals. (*C*) Intensity of ALP positive colonies per well. (*D*, *E*) PPA increased osteoblastic cell differentiation markers Col‐1 and OPN. (*F*) PPA suppressed adipogenesis evaluated by Oil Red‐O staining of ST2 cells after 6 days treatments with different doses of PPA (triplicates) in the presence of adipogenic medium, and inhibited adipocyte‐like cell differentiation. (*G*) Intensity of Oil Red‐O–stained adipocyte‐like cells per well. mRNA expression of genes adipsin (*H*) and CEBPα (*I*) after 1 day of treatment. Data are expressed as mean ± SE from triplicates of cell treatments (*n* = 3/group). Gene expression was relative to housekeeping gene GAPDH. Significant differences indicated by *p* < 0.05, *compared to control vehicle treatment. PPA = 3‐(3‐hydroxyphenyl)‐propionic acid; ALP = alkaline phosphatase; Col‐1 = collagen‐1; OPN = osteopontin; CEBPα = CCAAT/enhancer binding protein alpha; OB = osteogenic media; AD = adipogenic media.

We have studied the in vitro osteoblastogenic promoting properties of PPA at concentration ranges found in rat serum after consumption of a BB‐containing diet.^8^ The molecular structure of PPA is presented (Fig. 1 *A*). Our intent was to determine if PPA at the range of concentrations found in serum of rats fed a BB diet can promote osteoblastic cell differentiation. ST2 cells were cultured in osteogenic medium (OB) treated with four different concentrations of PPA. The concentrations ranged from 0.1 × to 100 × of the levels found in rat serum, where 1 × (10 µg/dL) represented the level found in serum of rats fed blueberry at 10% of calories.^8^ After 10 days of treatment, ALP staining, a well‐known assessment for osteoblast differentiation, showed increased osteoblastogenesis in 0.1 × to 10 × PPA‐treated cells (Fig. 1 *B*, *C*). Figure 1 *C* shows increased ALP‐positive colonies in PPA‐treated wells. RNA was collected following 24‐hour treatment with PPA from a separate experiment, and real‐time PCR analysis showed increased mRNA expression of osteoblast differentiation markers such as collagen‐1 (Col‐1) and osteopontin (OPN) in cells treated with PPA (Fig. 1 *D*, *E*). These are consistent with the ALP staining results. PPA was further tested for its ability to inhibit adipogenesis in cultures of pluripotent ST2 cells. ST2 cells were pretreated with four different concentrations of PPA for 24 hours before adding adipogenic medium. Oil red O staining indicated suppressed adipogenesis by PPA (Fig. 1 *F*, *G*). Figure 1 *G* shows decreased intensity of Oil red O–stained adipocyte‐like cells per well. Real‐time PCR analysis showed decreased mRNA expression of adipocyte‐like cell differentiation markers such as adipsin and CCAAT/enhancer binding protein (CEBPα) in cells treated with PPA (Fig. 1 *H*, *I*). These stimulatory and inhibitory effects of PPA on cell differentiation tended to be dose‐dependent in ST2 cells.

### PPA stimulates ^35^S‐GTPγS binding to GPR109A and inhibits cellular senescence

**Figure 2:**
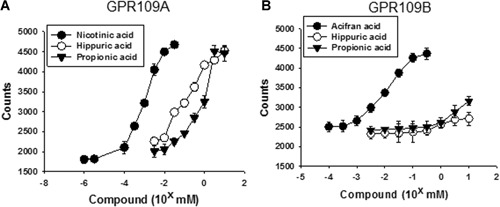
HA and PPA stimulate ^35^S‐GTPγS binding to membranes from mouse osteoblastic calvarial cells transfected with GPR109A but not GPR109B. Ligand‐induced ^35^S‐GTPγS binding was studied using membranes prepared from mouse fetal calvarial cells expressing mouse GPR109A (*A*) or GPR109B (*B*). Binding of ^35^S‐GTPγS was determined in the presence of increased concentration of nicotinic acid, acifran, HA, or PPA. HA = hippuric acid; PPA = 3‐(3‐hydroxyphenyl)‐propionic acid.

PPA and HA are phenolic acids and structurally similar to nicotinic acid. Assays for PPA and HA binding to G protein coupled receptor 109A (GPR109A, HM74a/PUMA‐G), the receptor for nicotinic acid were carried out. Ligand‐stimulated guanine nucleotide exchange using a ^35^S‐GTPγS binding assay was assessed, with membrane fractions prepared from mouse calvarial cells transfected with vectors that express either GPR109A or GPR109B. GPR109B is a close homolog of GPR109A with 95% amino acid sequence identity. Similar to nicotinic acid, PPA and HA bound to membranes from cells transfected with GPR109A (Fig. 2 *A*). As a positive control, we used acifran, a nicotinic acid analog known to be active on both GPR109A and GPR109B. Unlike acifran, we observed a very weak binding for both PPA and HA to GPR109B (Fig. 2 *B*).

**Figure 3:**
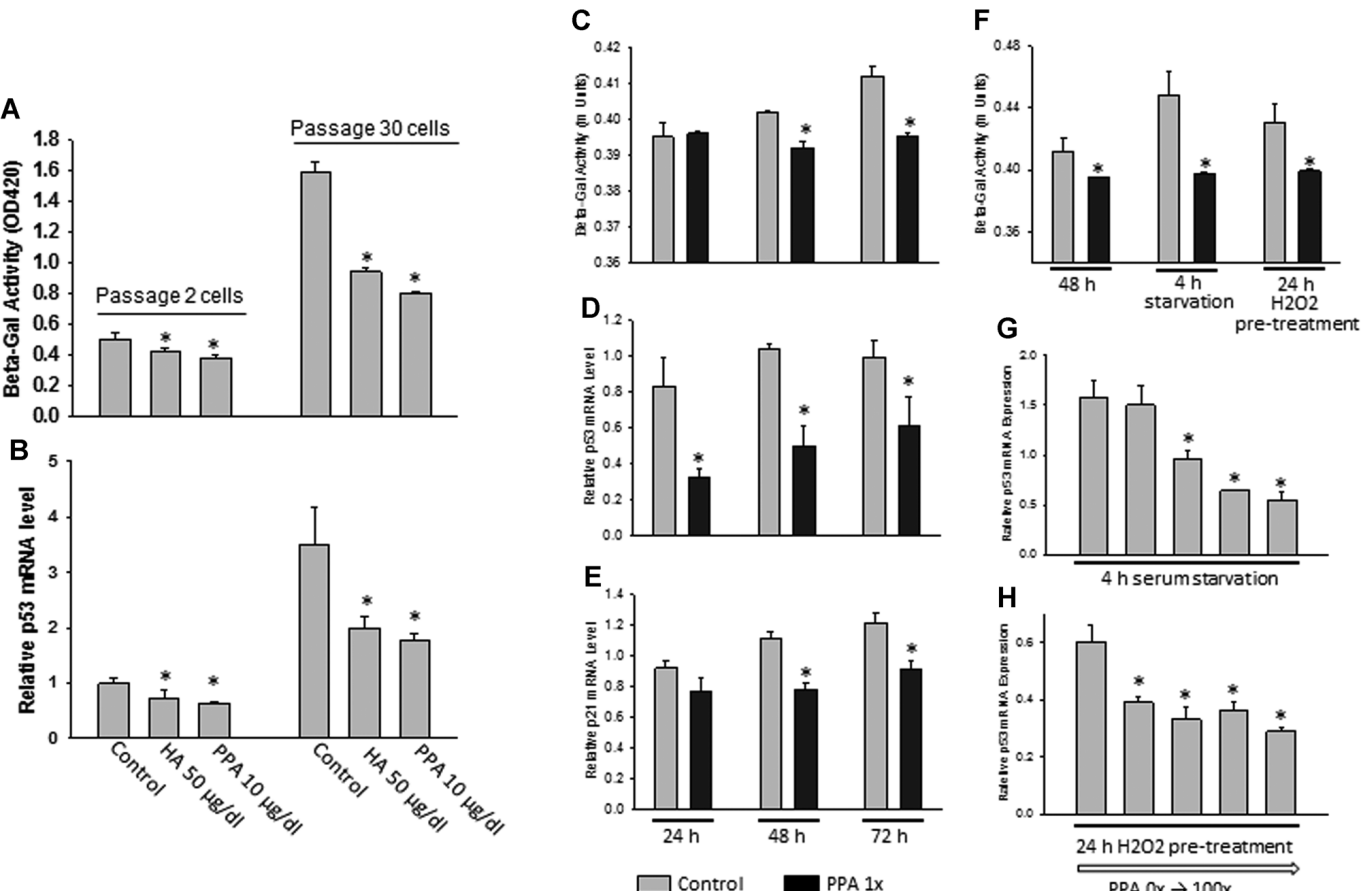
HA and PPA inhibit cellular senescence in a replicative senescent cell model by passaging mouse ST2 mesenchymal stem cells. (*A*, *B*) Senescence‐associated Beta‐Gal activity and p53 mRNA expression in passage 2 and passage 30 ST2 cells in the presence or absence of HA or PPA. PPA (1×) inhibits Beta‐Gal activity (*C*) in ST2 cells after 2 days (48 hours) and 3 days (72 hours), and inhibits p53 (*D*) and p21 (*E*) gene expression. (*F*) PPA inhibits serum starvation–induced Beta‐Gal activity in ST2 cells. PPA inhibits p53 gene expression after 4 hours of serum starvation (*G*), and 24‐hour H_2_O_2_ pretreatment (*H*). Data are shown as the means ± SE of triplicates of a representative experiment; gene expression was relative to housekeeping gene GAPDH. *p < 0.05 versus control. Beta‐Gal = beta‐galactosidase; HA = hippuric acid; PPA = 3‐(3‐hydroxyphenyl)‐propionic acid.

We next tested whether PPA can inhibit bone marrow stromal cell senescence; we have reported this action by HA previously.^9^ We have established a replicative senescent cell model by passaging mouse ST2 mesenchymal stem cells (MSCs) 30 times.^26^ Cells were treated with 1 × HA or PPA for 24 hours, and cell RNA and protein were extracted for real‐time PCR and senescence‐associated β‐galactosidase (Beta‐Gal) activity analysis following the first and last (30th) passages. HA and PPA significantly inhibited Beta‐Gal activity in first passage cells, and substantially inhibited Beta‐Gal activity in cells passaged 30 times with continuous treatments of HA and PPA (Fig. 3 *A*). In accordance with these data, significant downregulation of p53 gene expression was found in both the first and 30th passaged cells after treatment with HA or PPA compared to those without treatment (Fig. 3 *B*). To illustrate further of inhibitory effect of PPA on MSC senescence, ST2 cells were treated with 1 × of PPA for 3 days. Beta‐Gal activities in ST2 cells increased by time (Fig. 3 *C*), PPA inhibited Beta‐Gal activities on day 2 and day 3 (Fig. 3 *C*). Inhibitory effects of PPA on Beta‐Gal activities were accompanied by inhibition of p53 and p21 gene expression in ST2 cells (Fig. 3 *D*, *E*). Moreover, PPA also inhibited serum starvation and 25 µM hydrogen peroxide (H_2_O_2_)‐induced Beta‐Gal activities (Fig. 3 *F*). ST2 cells were serum starved for 4 hours or pretreated with H_2_O_2_ for 24 hours, although it did not show clear dose‐dependent effects, PPA inhibited p53 gene expression in those cells treated with serum starvation or H_2_O_2_ pretreatment (Fig. 3 *G*, *H*).

### PPA stimulates bone formation in rapidly growing mice

To better understand how different doses of PPA work on bone formation in vivo, we administered four doses of PPA (mice were injected i.p. with 0.1, 0.5, 1, and 5 mg/kg/day) to 1‐month‐old female C57BL6/J mice for 30 days. µCT scan on mouse tibias revealed that PPA, specifically at doses of 0.1 to 1 mg/kg/day, significantly increased trabecular bone acquisition (Table 1). At 6 weeks old, mice are still in the rapidly growing phase, and trabecular bone accumulation is close to maximal levels by this time. Treatment of mice with 0.1, 0.5, and 1 mg/kg/day PPA significantly increased trabecular bone volume (BV/TV), and connective density compared to those in control diet with saline treatment (Table 1). These increases in bone acquisition were characterized by significantly elevated trabecular thickness (Tb.Th), and significant decreases in trabecular spacing (Tb.Sp) in 0.1 mg/kg/day and 1 mg/kg/day PPA‐treated mice compared to control. As not to our expectation, trabecular number (Tb.N) did not show significant differences. There were no significant differences in cortical parameters among all groups with or without PPA treatments (Table 1).

**Table 1:** Trabecular and Cortical µCT Parameters on Tibias of Control or PPA‐Treated Mice

	Female mice PPA (mg/kg/day)				
	0	0.1	0.5	1	5
Trabecular					
BV/TV	0.18 ± 0.008	0.21 ± 0.005*	0.20 ± 0.006*	0.20 ± 0.006*	0.20 ± 0.009
Connectivity density	209.5 ± 23.4	259.8 ± 26.6*	234.1 ± 9.6*	229.9 ± 14.0*	239.3 ± 16.8*
SMI	1.98 ± 0.04	1.64 ± 0.06*	1.64 ± 0.08*	1.80 ± 0.1	1.73 ± 0.06*
Tb.N	4.91 ± 0.24	5.14 ± 0.12	4.83 ± 0.16	5.02 ± 0.07	4.89 ± 0.11
Tb.Th	0.049 ± 0.0003	0.051 ± 0.0007*	0.055 ± 0.002*	0.053 ± 0.002*	0.053 ± 0.0009*
Tb.Sp	0.21 ± 0.011	0.20 ± 0.006*	0.21 ± 0.01	0.20 ± 0.003*	0.21 ± 0.005
vBMD	719.8 ± 10.2	681.3 ± 8.4*	690.7 ± 6.0*	700.1 ± 5.0	713.2 ± 9.6
Cortical					
Cortical CSA	0.198 ± 0.004	0.189 ± 0.004	0.197 ± 0.004	0.197 ± 0.005	0.20 ± 0.004
Cortical thickness	0.164 ± 0.003	0.162 ± 0.003	0.161 ± 0.005	0.163 ± 0.002	0.167 ± 0.003
Total CSA	0.40 ± 0.008	0.38 ± 0.007	0.40 ± 0.01	0.40 ± 0.02	0.40 ± 0.01
Periosteal perimeter	1.50 ± 0.02	1.46 ± 0.02	1.55 ± 0.07	1.50 ± 0.04	1.51 ± 0.03
Midshaft diameter	0.52 ± 0.005	0.51 ± 0.006	0.50 ± 0.01	0.52 ± 0.01	0.52 ± 0.006
Medullary area	0.17 ± 0.006	0.16 ± 0.004	0.17 ± 0.009	0.17 ± 0.01	0.17 ± 0.01
Endosteal perimeter	0.99 ± 0.02	0.95 ± 0.02	1.04 ± 0.07	0.99 ± 0.03	0.97 ± 0.04

µCT measurements of trabecular and cortical of the tibial bone were evaluated using a Scanco µCT scanner (CT‐40; Scanco Medical AG, Bassersdorf, Switzerland) at 6 m isotropic voxel size with an X‐ray source at power 55 kV, current 145 A, and integration time 300 ms.

PPA = 3‐(3‐hydroxyphenyl) propionic acid; vBMD = volumetric bone mineral density.

* *p* < 0.05 versus 0 mg/kg/d, *n* = 10.

**Figure 4:**
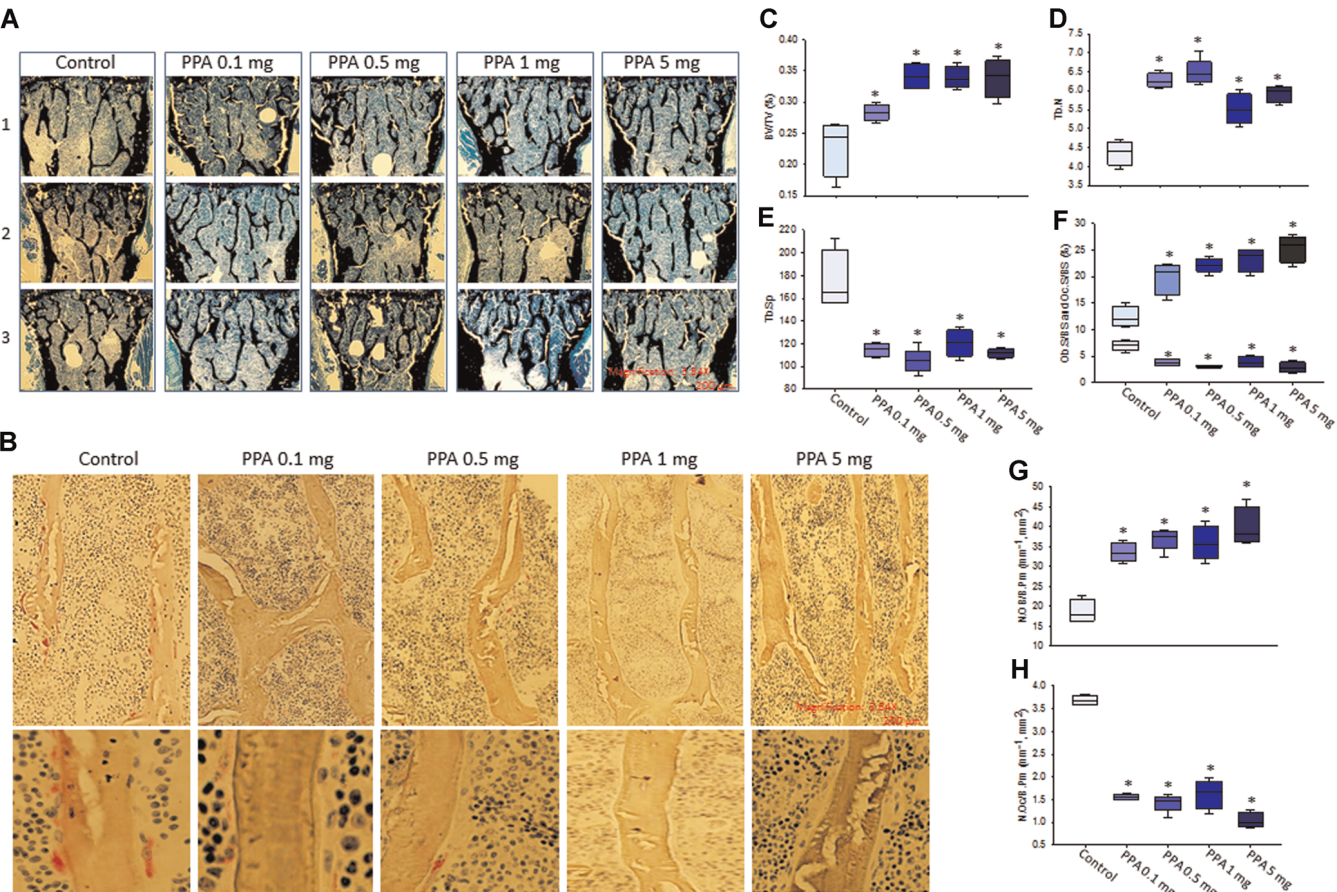
Histomorphometry and histology on L_5_ vertebrae shows increased bone formation in PPA‐treated mice. (*A*) Von Kossa staining on L_5_ vertebrae for histomorphometric analysis. Pictures represent three (1, 2, 3) individual mouse per each group, control, PPA 0.1 mg/kg/d (0.1 mg) to 5 mg/kg/d (5 mg) treatments, black lines show trabecular bone. Magnification 3.84 × , 200 µm, 4 × /0.13 PhL lens. (*B*) TRAPase staining on L_5_ vertebrae, pictures show one mouse per each group, red dots are TRAPase‐positive osteoclastic cells. Upper part pictures, magnification × 3.84, 200 µm, 20 × /0.4 Phc lens; lower part pictures, magnification 3.84 × , 200 µm, 40 × /0.55 Ph2 lens. Parameters from histomorphometric analysis, (*C*) BV/TV (%); (*D*) Tb.N; (*E*) Tb.Sp; (*F*) Ob.S/BS and Oc.S/BS; (*G*) N.OB/B.Pm; (*H*) N.OC/B.Pm. Data bars are expressed as mean ± SE (*n* = 5/group). Significant differences indicated by *p* < 0.05, *compared to control group. BV/TV = bone volume per total volume; Tb.N = trabecular number; Tb.Sp = trabecular separation; Ob.S/BS = osteoblast surface per bone surface; Oc.S/BS = osteoclast surface per bone surface; N.OB/B.Pm = number of osteoblast per bone parameter; N.OC/B.Pm = number of osteoclast per bone parameter; TRAPase = tartrate‐resistant acid phosphatase; PhL = Phase L; Phc = Phase C; Ph2 = Phase 2.

Histology and histomorphometric analysis were performed on mouse lumbar vertebrae (L_5_). Von Kossa staining showed increased trabecular bone in all PPA‐treated animals compared to control animals (Fig. 4 *A*, showing three animals per each treatment). TRAPase staining showed less pink‐stained osteoclastic cells in all PPA‐treated animals compared to control animals (Fig. 4 *B*). Parameters from histomorphometric read on lumbar vertebrae (L_5_) are slightly different than data from µCT on tibia. We found significantly increased BV/TV (%) (Fig. 4 *C*) and Tb.N (Fig. 4 *D*) in all PPA‐treated groups compared to control group, but there are no clear dose‐dependent effects. On the other hand, Tb.Sp significantly decreased in all PPA‐treated animals compared to control (Fig. 4 *E*). Ob.S/BS tended to increase dose‐dependently (Fig. 4 *F*), Oc.S/BS significantly decreased in all PPT‐treated groups compared to control group (Fig. 4 *F*) and this is consistent with TRAPase staining presented in Fig. 4 *B*. In accordance with these data, N.Ob increased in all PPA‐treated animals (Fig. 4 *G*); on the other hand, N.Oc significantly decreased in PPA‐treated groups compared to control group (Fig. 4 *H*).

Serum bone‐specific ALP levels, a bone formation marker, and total osteocalcin (Gla‐OC) levels were increased significantly in all PPA‐treated groups compared to saline control (Table 2). Increased bone formation markers in all PPA‐treated animal groups did not exactly reflect dose‐associated µCT bone parameters, but qualitatively were consistent with an osteoblast‐stimulating phenotype and histomorphometric data from vertebrae. ALP and osteocalcin levels were also measured in bone marrow plasma. Bone formation markers ALP and total osteocalcin levels in bone marrow plasma were significantly increased in all PPA‐treated groups compared to control, but there was no dose‐dependent trend (Table 2). In bone marrow plasma, concentrations of undercarboxylated osteocalcin, which is an active form of osteocalcin, were found significantly higher in only 0.1 mg/kg/day and 0.5 mg/kg/day PPA‐treated groups compared to saline control group (Table 2).

**Table 2:** Bone Formation Markers in Serum and Bone Marrow Plasma of Control or PPA‐Treated Mice

	Female mice				
	0	0.1	0.5	1	5
Serum					
ALP (µUnits/min)	0.529 ± 0.017	0.596 ± 0.0.024*	0.596 ± 0.029*	0.593 ± 0.015*	0.581 ± 0.019*
Gla‐OC (ng/mL)	83.1 ± 24.4	147.4 ± 23.1*	176.7 ± 8.5*	177.9 ± 9.0*	176.4 ± 13.0*
Bone marrow plasma					
ALP (µUnits/min)	0.269 ± 0.026	0.489 ± 0.067*	0.444 ± 0.059*	0.356 ± 0.050*	0.580 ± 0.139*
Gla‐OC (ng/mL)	69.8 ± 4.3	89.8 ± 8.0*	84.1 ± 6.1*	87.7 ± 2.7*	90.3 ± 4.5*
Glu‐OC (ng/mL)	13.43 ± 0.90	36.99 ± 8.23*	27.02 ± 3.91*	16.57 ± 1.80	15.90 ± 1.98

PPA = 3‐(3‐hydroxyphenyl) propionic acid; Gla‐OC = total osteocalcin; Glu‐OC = undercarboxylated osteocalcin; EIA = enzyme immunoassay.

* *p* < 0.05 versus 0 mg/kg/day, *n* = 10; EIA kit from Takara Bio.

**Figure 5:**
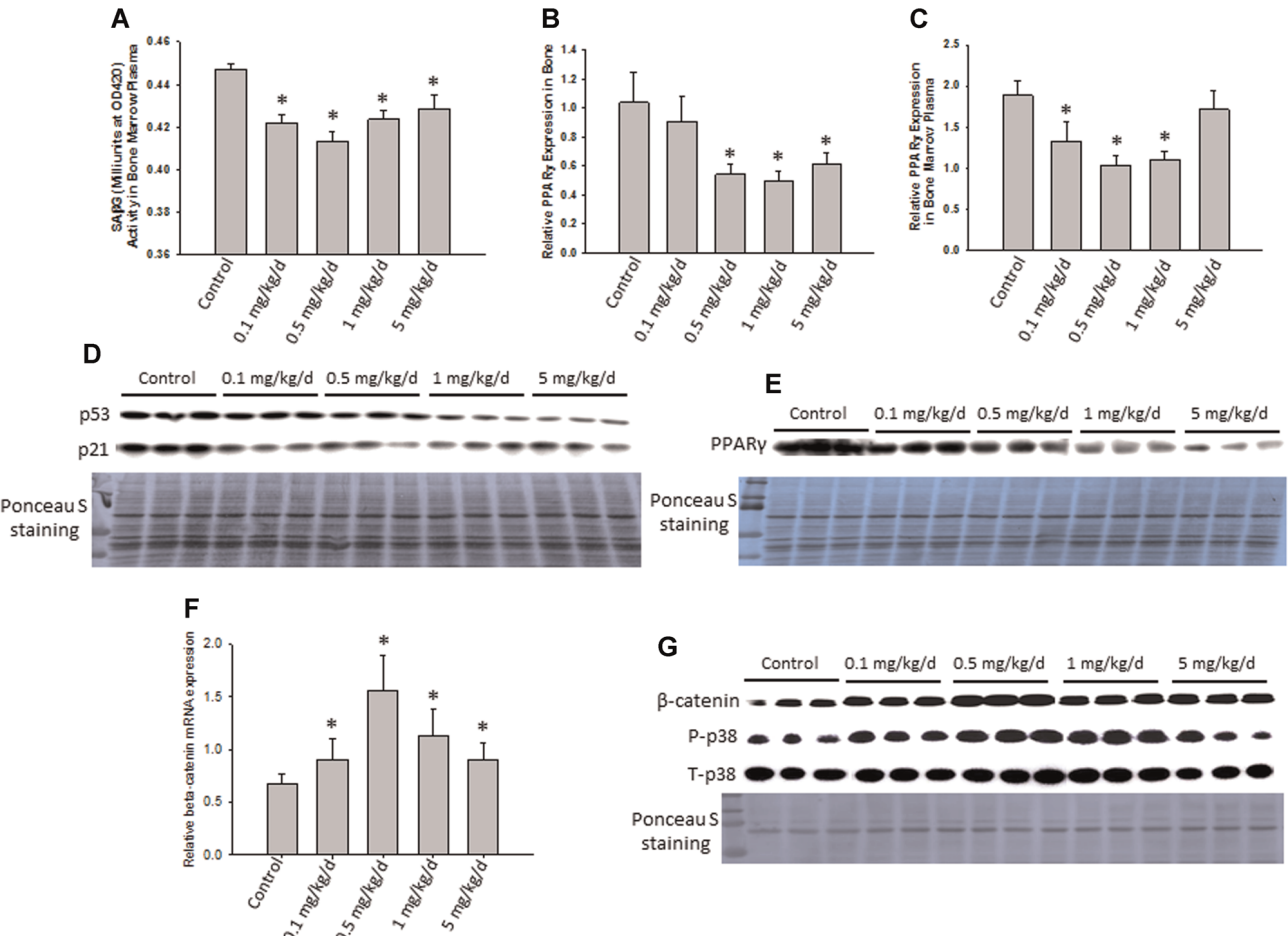
PPA suppresses senescence signaling in bone from in vivo young mice. (*A*) Proteins were isolated from L_4_ vertebrae specifically for senescence marker SAβG (Beta‐Gal) activity measurement. (*B*, *C*) Total RNA were also isolated from L_4_ vertebrae and precipitated bone marrow after spinning, real‐time PCR was used for determining PPARγ gene expression in all groups of mice. (*D*, *E*) Proteins were isolated from L_3_ vertebrae of all groups of mice, and samples were pooled to three samples per group for Western blots p53/p21 and PPARγ protein expression. Ponceau S staining is shown for protein loading controls. (*F*) Real‐time PCR was used for determine β‐catenin mRNA expression in all groups of mice. (*G*) Western blots for b‐catenin and p38 protein expression in all group of mice. Data bars are expressed as mean ± SE (*n* = 10/group). Significant differences indicated by *p* < 0.05, *compared to control animals. SAβG = senescence‐associated β‐galactosidase; Beta‐Gal = beta‐galactosidase.

We isolated proteins and total RNA from L_4_ vertebrae for measurement of senescence‐associated β‐galactosidase (Beta‐Gal) activity in bone. Western blot and real‐time PCR analysis were performed to determine possible bone‐forming signal transduction pathways. All four PPA treatments significantly inhibited Beta‐Gal activity with the 0.5‐mg/kg/day dose having the greatest inhibition (Fig. 5 *A*). PPARγ expression in total RNA from both bone (Fig. 5 *B*) and bone marrow plasma (Fig. 5 *C*) was downregulated in both 0.5 mg/kg/day and 1 mg/kg/day PPA‐treated animals compared to saline control. In accordance with these data, Western blots showed downregulation of PPARγ protein expression in whole bone after PPA treatments (Fig. 5 *E*), with the protein expression pattern relatively matching its mRNA pattern. P53 protein expression in bone was also downregulated by PPA treatments, especially at the higher doses (Fig. 5 *D*), indicating an anti‐senescence property of PPA in bone. Moreover, β‐catenin mRNA expression in bone were significantly increased from all PPA‐treated animal groups compared to control group (Fig. 5 *F*). This is consistent with its protein expression and phosphor‐p38 (p‐p38) expression (Fig. 5 *G*).

### Changes in gene expression in bone in response to PPA treatment

**Figure 6:**
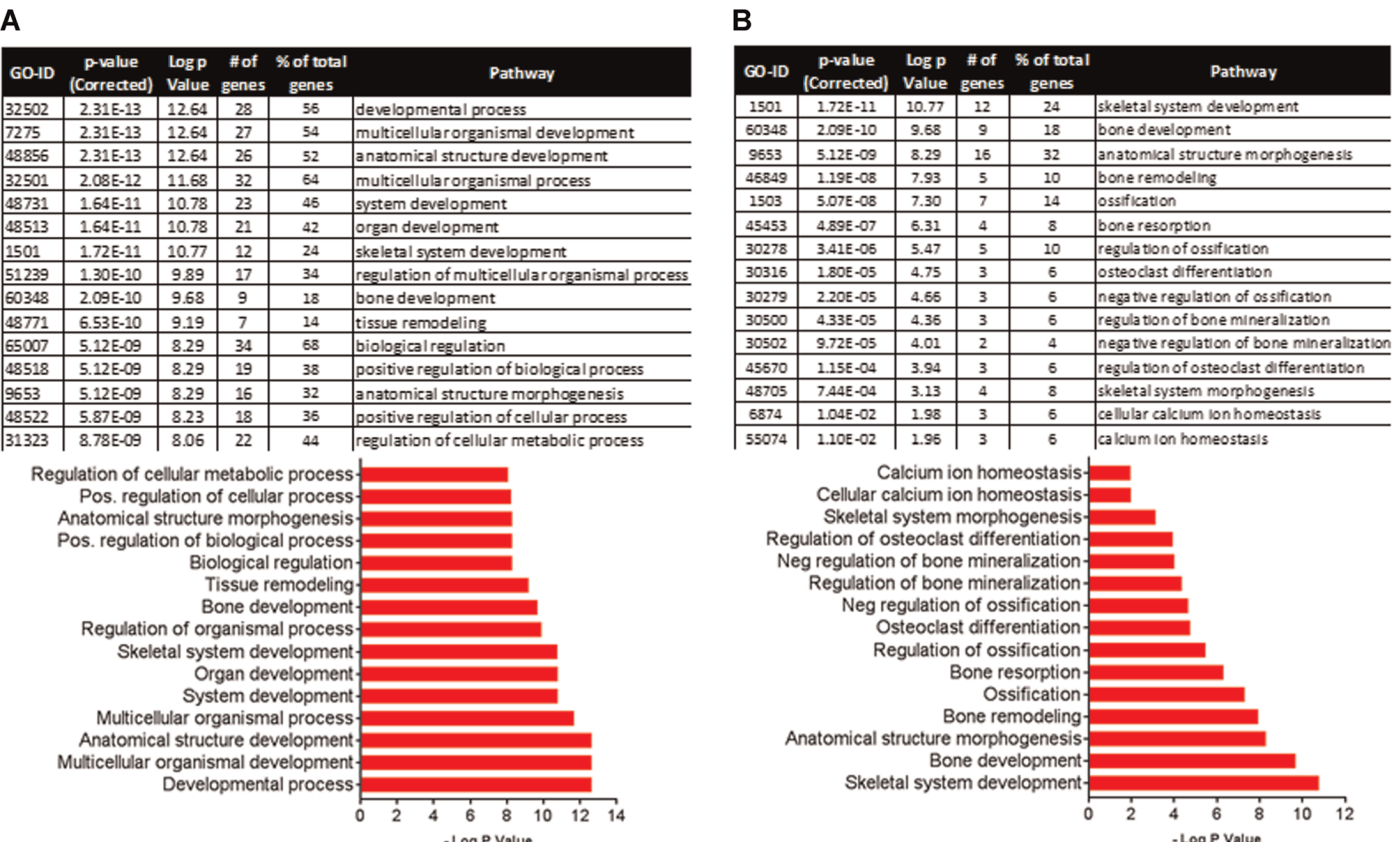
Pathway analysis of 50 genes involved in bone metabolism from RNA‐sequencing data of control and PPA‐treated (0.5 mg/kg/day) animals with at least a 1.5‐fold change in expression. (*A*) Top 15 overall enriched pathways: 68% (34 genes); 64% (32 genes); and 56% (28 genes) are the top three groups of genes involved in biological regulation, multicellular organismal process, and development process, respectively. (*B*) Top 15 bone related pathways: 32% (16 genes) and 24% (12 genes) are involved in anatomical structure morphogenesis and skeletal system development, respectively.

To identify additional genes in bone that are responsive to PPA treatment, we carried out RNA‐seq–based gene expression profiling. We found that bone‐specific genes collagen 1, osteocalcin (bone gamma‐carboxyglutamate [gla] protein), and osteonectin were among the top 50 highly expressed genes based on normalized reads per kilobase of transcript per million mapped (RPKM) values, indicating that the RNA preparations were, as expected, enriched with bone tissue. Differential expression analysis of all genes showed that PPA treatment affected expression of 158 transcripts (±2‐fold change, *p* < 0.05) (Supporting Table 1). Gene ontology analysis using BiNGO showed that small molecule signaling, including amine metabolism and oxoacid metabolism, were significantly influenced by PPA treatment (Supporting Table 2). Further, predominant changes in genes involved in acute stimulus response and inflammatory response were also affected. In this context, mRNA expression of neutrophil elastase, myeloperoxidase, and neutrophil granule protein (Npg) were downregulated. To examine the influence of PPA treatment within genes involved in skeletal functions, we identified 550 bone, ossification, osteoclast, osteoblast, and skeletal‐associated genes using gene ontology definitions. Among these, 50 genes showed a minimum ±1.5‐fold change in expression in PPA‐treated animals compared to control animals. These 50 genes were analyzed to determine which biological pathways were affected by PPA treatment. Of the top 15 pathways, biological regulation, multicellular organismal process, and development process were the top three groups in terms of number of genes involved at 68% (34 genes), 64% (32 genes), and 56% (28 genes), respectively (Fig. 6 *A*). Moreover, focusing on pathways specific to bone, anatomical structure morphogenesis (32%, 16 genes), bone development (18%, 9 genes), and skeletal system development (24%, 12 genes) were the top three pathways enriched (Fig. 6 *B*). These data are consistent with the proposal that PPA is involved in promotion of bone development.

**Figure 7:**
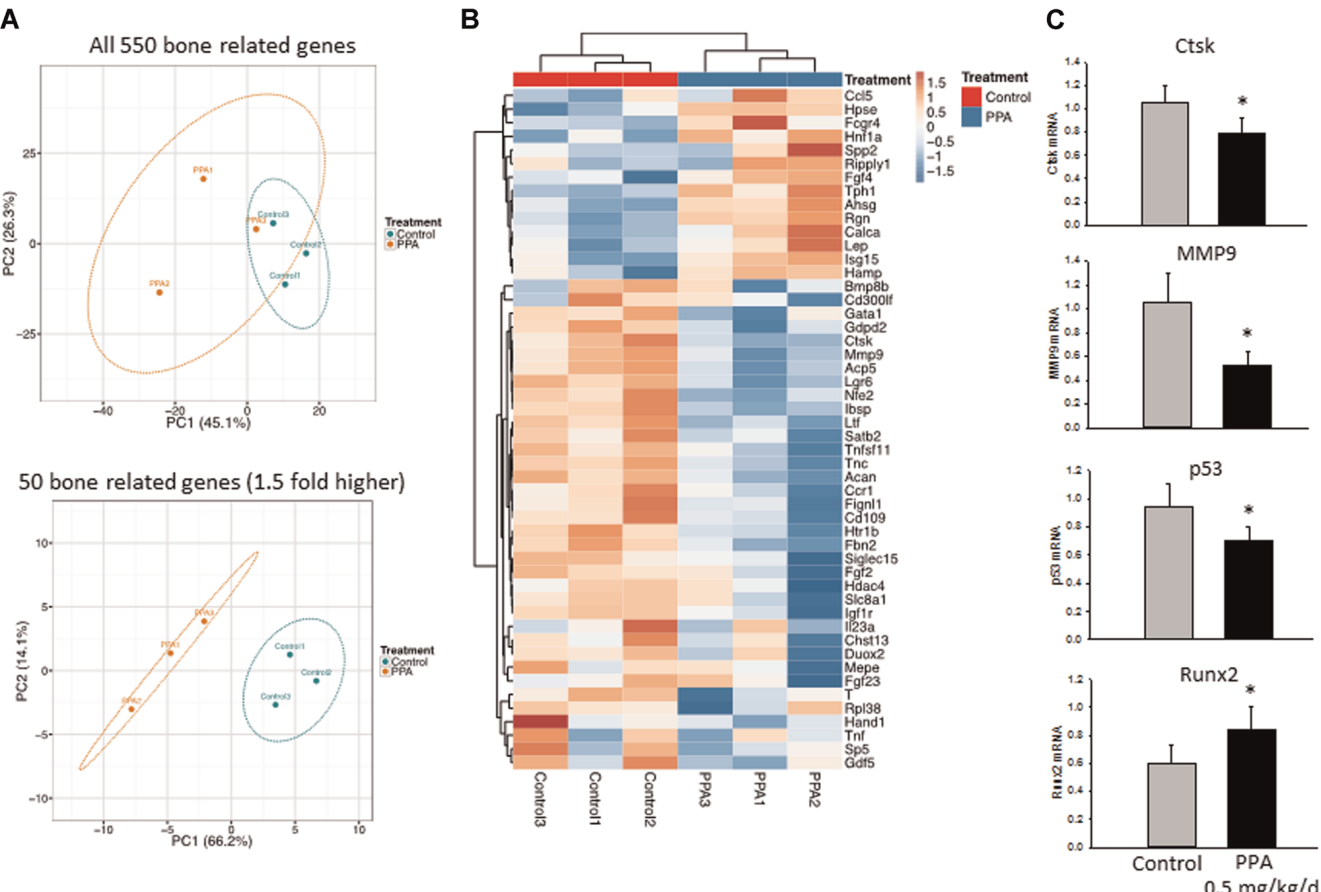
RNA‐sequencing data of significantly fold‐changed genes possibly involved in bone formation or resorption in bone between two treatment groups. (*A*) Principal component analysis of predicted functional metagenomics pathways of all 550 bone‐related genes and 1.5‐fold changed 50 bone‐related genes using the PICRUSt bioinformatics software package. (*B*) Heat map of 1.5‐fold changed 50 bone‐related genes representing three pools (control 1, 2, 3 versus 0.5 mg/kg/day PPA treatment 1, 2, 3). (*C*) Real‐time PCR confirms gene expression of Ctsk and MMP9 (plus p53 and Runx2 were not included in the list) between two groups. Significant differences indicated by *p* < 0.05, *0.5 mg/kg/day PPA treatment compared to either control. Ctsk = cathepsin K.

Principal component analysis of gene expression profiles showed that subsets of transcripts discriminated control and PPA samples, indicating a significant effect of PPA on bone gene expression (Fig. 7 *A*). Those 50 genes that had at least 1.5‐fold changes in their expression were further analyzed and presented in heat map (Fig. 7 *B*). Using real‐time PCR, we found that PPA treatment in vivo not only suppressed expression of genes that are involved in senescence pathway, such as p53 and MMP9, but also increased expression of genes involved in osteoblast differentiation, such as Runx2 (Fig. 7 *C*). Surprisingly, PPA treatment significantly downregulated expression of genes involved in osteoclastogenesis or bone resorption (one of the pathways listed in Fig. 7 *B*), such as cathepsin k (Ctsk) (Fig. 7 *C*), suggesting that PPA inhibits bone resorption and/or modifies osteoclast function in mice.

## Discussion

In the current report, we present in vitro and in vivo results of PPA effects on osteoblastic cell activity and bone accrual in young mice. This compound (unconjugated free form) was found to be the second most abundant phenolic acid among the phenolic acids that had at least a 10‐fold higher concentration in the serum of rats fed a BB‐containing diet compared to those fed a control diet.^8^ Based on transcript and protein expression patterns, we propose that the cellular and skeletal effects of this compound are mediated in part through activation of osteoblast differentiation and activity, reduction in osteoblast cell senescence pathways, and possibly through suppression of PPARγ‐mediated adipogenesis from MSCs, thus favoring osteoblastogenesis. Although the optimal dose of PPA needed to increase net bone formation in animal models still needs to be fully verified, mechanisms of the beneficial effect of PPA on the skeletal system may be similar to HA^8^ and other phenolic acids that have been described previously.^27^

Nutritional research on bone health indicates the importance of an adequate intake of dietary calcium, vitamin D, protein, and phytochemicals. Research into the correlation between fruit and vegetable intake and bone mineral density has suggested a role for polyphenolic compounds found in fruits and vegetables in promoting bone health.^28^ However, there have been few mechanisms confirmed, and a dearth of specific compounds under investigation as potential dietary supplements or for long‐term therapeutic alternatives to chemically‐synthesized medicines for boosting peak bone formation. For reasons and mechanisms not well understood, previous reports on phenolic acids’ bone protective, antioxidant, and anti‐inflammatory effects have been highly variable: different phenolic acids may have different or opposite effects on osteoblast differentiation or on decreasing the formation of osteoclast‐like cells.^29^ For example, chlorogenic and caffeic acids were previously observed to negatively regulate osteoblast differentiation as shown by dose‐dependent impacts on reducing ALP activity.^27^

In our current report, we performed cell culture studies demonstrating that PPA enhanced preosteoblast differentiation while inhibiting adipogenesis in common progenitor cells. This finding suggested that PPA is capable to drive stem cell differentiation potential, and may have a profound impact on bone health promotion. Low‐dose PPA had convincing effects on promoting osteoblastogenesis and gene expression, suggesting differentiation of MSCs toward tissue specific lineages, for example to either osteoblasts or adipocytes, is sensitive to environmental conditions. We suspect very little changes of bone metrics, or plasticity or local acid‐base balance^30^, ^31^ by PPA may alter stem cell differentiation program.

Circulating PAs such as PPA are gut microflora–derived metabolites of polyphenols following consumption of BB and other fruits, vegetables, and coffee.^32^ Most PAs are structurally similar to nicotinic acid or niacin (the essential nutrient, vitamin B3), the nicotinic acid metabolite that binds and perhaps activates the G‐protein coupled receptor GPR109A.^33^ PPA and HA have a high affinity to bind GPR109A, but are largely inactive on its homologous receptor GPR109B, consistent with previous observations.^11^ We therefore suspected that the actions of PAs on bone cells may be mediated through mechanisms involving activation of GPR109A, although the role of GPR109A itself in skeletal development is unknown. GPRs constitute the largest family of cell‐surface molecules involved in signal transduction and cell differentiation, and have emerged as crucial players in child development, growth, and maturation.^34^ They are targets for many approved drugs. GPRs are capable of recruiting and regulating the activities of specific heterotrimeric G proteins, which are specialized signal transducers composed of three subunits: α, β, and γ. Secondary messenger cAMP is usually involved in G protein receptor signaling, which is known to be important in many aspects of cell physiology, including cell growth, survival, and differentiation.^35^ Several subtypes of GPRs have been shown to be involved in skeletal growth^36^ and obesity development. Future studies that leverage GPR109A knockout mice should help untangle whether GPR109A plays a role in the pathway of osteoblast or adipocyte and osteoclast differentiation, and whether PPA or HA truly promote osteoblastogenesis through GPR109A.

Bone mass accumulations during puberty (ages 12 to 18 years) are substantial, and fast bone remodeling favors osteoblastic bone formation over osteoclast bone resorption. Recent studies in animal models have shown that sufficient or optimal bone formation during early life is critical for delaying bone loss during aging, and such bone formation during early life can be epigenetically regulated.^37^ For that reason, we chose a young rapidly growing mouse model to study PPA in vivo, aiming to see whether PPA promotes bone growth during early life and whether the effect could persist into adulthood. The in vivo effects of PPA on bone formation indicates that 0.1 mg/kg/day to 0.5 mg/kg/day PPA clearly increased trabecular bone formation in female mice, among several other significant effects. These are not only supported by measurements of bone turnover markers in both serum and bone marrow plasma, and increased expression of osteoblast differentiation marker Runx2, but also by a decrease in gene expression such as PPARγ in bone and bone marrow. Our data implicated that the effects of PPA on bone properties as revealed by µCT (Table 1) and histomorphometry (Fig. 4) are different. It appears that the effects on vertebrate are more prominent than that on the tibia. µCT is based on radiological properties of the skeleton whereas histomorphometry allows cellular quantification of bone cell subtypes and is based on manual tracing on histological slides. Hence, a number of internal inconsistencies could be due to the specifics of the methods and the surrogates they represent. Because the compound was subcutaneously injected, as designed, it is completely possible; however, our current studies are not able to address whether the compound PPA has different effects in the vertebrae versus the tibia. Hence, we will apply µCT on both bone site to compare the effect of the compound, and subcutaneous injection versus oral administration in our future studies. Further, because the tibia is much more influenced by body weight in rodents relative to vertebrae, this could be an additional indirect confounder.

Moreover, consistent with cell culture data, tissue senescence marker Beta‐Gal activity measurement in bone marrow plasma suggests PPA has anti‐senescence properties. We used three different cell culture models, ie, physiological cellular senescence cell culture model, cell cultures with serum starvation, and hydrogen peroxide pretreatment to induce cellular senescence model, and examined that PPA clearly inhibits both physiological and pathological cellular senescence programs. The inhibitory effect of PPA on osteoblastic cell senescence signaling may offer some valuable guidance to the clinical practice. We were the first group previously to describe contribution of osteoblast senescence to ovariectomy‐induced bone loss;^14^ therefore, PPA may be considered as therapeutic compound to postmenopausal or aging‐induced osteoporosis on targeting osteoblasts to prevent their senescence program.

Interestingly, by using next‐generation RNA‐sequencing (RNA‐seq) analyses, we have shown that PPA not only increased expression of genes involved in skeletal development and bone mineralization, but also suppressed tissue senescence markers such as MMP9.^22^ Furthermore, PPA significantly reduced Ctsk gene expression, a specific osteoclast differentiation marker. Taken together with CTX data in animal serum, we believe that PPA may have a significant effect on suppressing bone resorption. This hypothesis awaits confirmation from future investigation.

In conclusion, we have characterized the effects of PPA on inhibiting senescence signaling to promote MSC differentiation potential toward osteoblasts, while suppressing adipogenesis. In an animal model of PPA treatment, we found that relatively low‐dose PPA resulted in significantly better bone volume and trabecular thickness compared to controls. PPA was capable of altering the MSC differentiation program and bone cell senescence. Thus, our findings lead us to propose a novel idea that diet‐derived PAs such as PPA and HA are capable of altering stem cell differentiation and senescence programming that is important to net bone formation, and that these effects may in part be through actions at GPR109A in bone cells.

## Disclosures

All authors state that they have no conflicts of interest.

## Supporting information

Supporting Information .

Supporting Information .

Supporting Information .

## References

[1] Kular J , Tickner J , Chim SM , Xu, J . An overview of the regulation of bone remodelling at the cellular level. Clin Biochem. 2012;45:863–3. 22465238 10.1016/j.clinbiochem.2012.03.021

[2] Zaidi M . Skeletal remodeling in health and disease. Nat Med. 2007;13:791–801. 17618270 10.1038/nm1593

[3] Ogita M , Rached MT , Dworakowski E , Bilezikian JP , Kousteni S . Differentiation and proliferation of periosteal osteoblast progenitors are differentially regulated by estrogens and intermittent parathyroid hormone administration. Endocrinology. 2008;149:5713–23. 18617606 10.1210/en.2008-0369 PMC2584601

[4] Tylavsky FA , Holliday K , Danish R , Womack C , Norwood J , Carbone L . Fruit and vegetable intakes are an independent predictor of bone size in early pubertal children. Am J Clin Nutr. 2004;79:311–17. 14749239 10.1093/ajcn/79.2.311

[5] Lanham SA . Fruit and vegetables: the unexpected natural answer to the question of osteoporosis prevention? Am J Clin Nutr. 2006;83:1254–55. 16762933 10.1093/ajcn/83.6.1254

[6] Setchell KD , Lydeking‐Olsen E . Dietary phytoestrogens and their effect on bone: evidence from in vitro and in vivo, human observational, and dietary intervention studies. Am J Clin Nutr. 2003;78(3 Suppl):593S–609S. 12936954 10.1093/ajcn/78.3.593S

[7] Tseng PC , Hou SM , Chen RJ , et al. Resveratrol promotes osteogenesis of human mesenchymal stem cells by upregulating RUNX2 gene expression via the SIRT1/FOXO3A axis. J Bone Miner Res. 2011;26:2552–63. 21713995 10.1002/jbmr.460

[8] Chen JR , Lazarenko OP , Wu X , et al. Dietary‐induced serum phenolic acids promote bone growth via p38 MAPK/β‐catenin canonical Wnt signaling. J Bone Miner Res. 2010;25:2399–2411. 20499363 10.1002/jbmr.137

[9] Chen JR , Lazarenko OP , Zhang J , Blackburn ML , Ronis MJ , Badger TM . Diet‐derived phenolic acids regulate osteoblast and adipocyte lineage commitment and differentiation in young mice. J Bone Miner Res. 2014;29:1043–53. 23832484 10.1002/jbmr.2034

[10] Herrmann K . Occurrence and content of hydroxycinnamic and hydroxybenzoic acid compounds in foods. Crit Rev Food Sci Nutr. 1989;28:315–47. 2690858 10.1080/10408398909527504

[11] Ren N , Kaplan R , Hernandez M , et al. Phenolic acids suppress adipocyte lipolysis via activation of the nicotinic acid receptor GPR109A (HM74a/PUMA‐G). J Lipid Res. 2009;50:908–14. 19136666 10.1194/jlr.M800625-JLR200 PMC2666177

[12] Rodan GA , Martin TJ . Therapeutic approaches to bone diseases. Science. 2000;289:1508–14. 10968781 10.1126/science.289.5484.1508

[13] McCulloch CA , Heersche JN . Lifetime of the osteoblast in mouse periodontium. Anat Rec. 1988;222:128–35. 3213963 10.1002/ar.1092220204

[14] Zhang J , Lazarenko OP , Blackburn ML , Badger TM , Ronis MJ , Chen JR . Blueberry consumption prevents loss of collagen in bone matrix and inhibits senescence pathways in osteoblastic cells. Age. 2013;35:807–20. 22555620 10.1007/s11357-012-9412-z PMC3636388

[15] Zhang J , Lazarenko OP , Blackburn ML , et al. Feeding blueberry diets in early life prevent senescence of osteoblasts and bone loss in ovariectomized adult female rats. PLoS One. 2011;6:e24486. 21912699 10.1371/journal.pone.0024486 PMC3166322

[16] Bouxsein ML , Boyd SK , Christiansen BA , Guldberg RE , Jepsen KJ , Müller R . Guidelines for assessment of bone microstructure in rodents using micro–computed tomography. J Bone Miner Res. 2010;25:1468–86. 20533309 10.1002/jbmr.141

[17] Cao JJ , Gregoire BR , Gao H . High‐fat diet decreases cancellous bone mass but has no effect on cortical bone mass in the tibia in mice. Bone. 2009;44:1097–1104. 19264159 10.1016/j.bone.2009.02.017

[18] Chen JR , Zhang J , Lazarenko OP , et al. Soy protein isolates prevent loss of bone quantity associated with obesity in rats through regulation of insulin signaling in osteoblasts. FASEB J. 2013;27:3514–23. 23776073 10.1096/fj.12-226464

[19] Chen JR , Lazarenko OP , Shankar K , Blackburn ML , Badger TM , Ronis MJ . A role for ethanol‐induced oxidative stress in controlling lineage commitment of mesenchymal stromal cells through inhibition of Wnt/beta‐catenin signaling. J Bone Miner Res. 2010;25:1117–27. 20200986 10.1002/jbmr.7 PMC3153370

[20] Chen JR , Zhang J , Lazarenko OP , et al. Inhibition of fetal bone development through epigenetic down‐regulation of HoxA10 in obese rats fed high‐fat diet. FASEB J. 2012;26:1131–41. 22131269 10.1096/fj.11-197822

[21] Chen JR , Lazarenko OP , Shankar K , et al. Inhibition of NADPH oxidases prevents chronic ethanol‐induced bone loss in female rats. J Pharmacol Exp Ther. 2011;336:734–42. 21098090 10.1124/jpet.110.175091 PMC3061541

[22] Chen JR , Lazarenko OP , Blackburn ML , et al. Maternal obesity programs senescence signaling and glucose metabolism in osteo‐progenitors from rat and human. Endocrinology. 2016;157:4172–83. 27653035 10.1210/en.2016-1408

[23] Thakali KM , Faske JB , Ishwar A , et al. Maternal obesity and gestational weight gain are modestly associated with umbilical cord DNA methylation. Placenta. 2017;57:194–203. 28864012 10.1016/j.placenta.2017.07.009

[24] Shankar K , Zhong Y , Kang P , et al. RNA‐seq analysis of the functional compartments within the rat placentation site. Endocrinology. 2012;153:1999–2011. 22355068 10.1210/en.2011-1833 PMC5393303

[25] Wankhade UD , Zhong Y , Kang P , et al. Enhanced offspring predisposition to steatohepatitis with maternal high‐fat diet is associated with epigenetic and microbiome alterations. PLoS One. 2017;12:e0175675. 28414763 10.1371/journal.pone.0175675 PMC5393586

[26] Zhang J , Lazarenko OP , Blackburn ML , Badger TM , Ronis MJ , Chen JR . Soy protein isolate down‐regulates caveolin‐1 expression to suppress osteoblastic cell senescence pathways. FASEB J. 2014;28:3134–45. 24719353 10.1096/fj.13-243659

[27] Folwarczna J , Pytlik M , Zych M , et al. Effects of caffeic and chlorogenic acids on the rat skeletal system. Eur Rev Med Pharmacol Sci. 2015;19:682–93. 25753887

[28] Horcajada MN , Offord E . Naturally plant‐derived compounds: role in bone anabolism. Curr Mol Pharmacol. 2012;5:205–18. 21787284 10.2174/1874467211205020205

[29] Islam MA , Alam F , Solayman M , Khalil MI , Kamal MA , Gan SH . Dietary phytochemicals: natural swords combating inflammation and oxidation‐mediated degenerative diseases. Oxid Med Cell Longev. 2016;2016:5137431. 27721914 10.1155/2016/5137431 PMC5046019

[30] Lee SG , Yang M , Wang Y , et al. Impact of orange juice consumption on bone health of the U.S. population in the national health and nutrition examination survey 2003‐2006. J Med Food. 2014;17:1142–50. 25055347 10.1089/jmf.2013.0072

[31] Hanley DA , Whiting SJ . Does a high dietary acid content cause bone loss, and can bone loss be prevented with an alkaline diet? J Clin Densitom. 2013;16:420–25. 24094472 10.1016/j.jocd.2013.08.014

[32] Williamson G , Clifford MN . Role of the small intestine, colon and microbiota in determining the metabolic fate of polyphenols. Biochem Pharmacol. 2017;139:24–39. 28322745 10.1016/j.bcp.2017.03.012

[33] Ahmed K , Tunaru S , Offermanns S GPR109A, GPR109B and GPR81, a family of hydroxy‐carboxylic acid receptors. Trends Pharmacol Sci. 2009;30:557–62. 19837462 10.1016/j.tips.2009.09.001

[34] Latronico AC , Hochberg Z . G protein‐coupled receptors in child development, growth and maturation. Sci Signal. 2010;3(143):re7. 20940428 10.1126/scisignal.3143re7

[35] Vinolo MA , Hirabara SM , Curi R . G‐protein‐coupled receptors as fat sensors. Curr Opin Clin Nutr Metab Care. 2012;15:112–16. 22234165 10.1097/MCO.0b013e32834f4598

[36] Mårtensson UE , Salehi SA , Windahl S , et al. Deletion of the G protein‐coupled receptor 30 impairs glucose tolerance, reduces bone growth, increases blood pressure, and eliminates estradiol‐stimulated insulin release in female mice. Endocrinology. 2009;150:687–98. 18845638 10.1210/en.2008-0623

[37] Chen JR , Lazarenko OP , Blackburn ML , Shankar K . Dietary factors during early life program bone formation in female rats. FASEB J. 2017;31:376–87. 27733448 10.1096/fj.201600703R

