# Supplementary material for: 3‐(3‐Hydroxyphenyl)‐Propionic Acid (PPA) Suppresses Osteoblastic Cell Senescence to Promote Bone Accretion in Mice
Source: JBMR Plus. 2019 Aug 23;3(9):e10201. doi: 10.1002/jbm4.10201 (PMC6808226; doi:10.1002/jbm4.10201)
Supplement: Supplementary file 2 — Supporting Information . [file JBM4-3-na-s002.docx]

Supplemental Table 2
